# Phylogenetic Groups, Virulence Factors, and Antimicrobial Susceptibility of *Escherichia coli* Associated with Urinary Tract Infections from a Metropolitan Area of Buenos Aires, Argentina

**DOI:** 10.3390/antibiotics15040350

**Published:** 2026-03-29

**Authors:** Nora B. Molina, Ramón A. González Pasayo, Marisa A. López, Mónica D. Sparo

**Affiliations:** 1 Centro Universitario de Estudios Microbiológicos y Parasitológicos (CUDEMYP, UNLP-CIC), Facultad de Ciencias Médicas, Universidad Nacional de La Plata, Calle 60 y 120 s/n, La Plata 1900, Buenos Aires, Argentina; 2 Instituto de Innovación para la Producción Agropecuaria y el Desarrollo Sostenible (IPADS, INTA- CONICET), Estación Experimental Agropecuaria Balcarce, Ruta 226 km 73.5, Balcarce 7620, Buenos Aires, Argentina; 3 Instituto de Investigación en Ciencias de la Salud, Facultad de Ciencias de la Salud, Universidad Nacional del Centro de la Provincia de Buenos Aires, Avda. Pringles 4375, Olavarría 7400, Buenos Aires, Argentina

**Keywords:** *Escherichia coli*, phylogenetic groups, virulence factors, antimicrobial susceptibility, biofilms, colistin resistance, multidrug resistance

## Abstract

**Background:** Uropathogenic *Escherichia coli* (UPEC) is the primary etiological agent of urinary tract infections (UTIs) worldwide. The emergence of strains combining high virulence with multidrug resistance (MDR) poses a significant challenge to public health. This study aimed to characterize the phylogenetic distribution, virulence profiles, and antimicrobial susceptibility of UPEC isolates recovered from patients in the metropolitan area of Buenos Aires (AMBA), Argentina. **Methodology:** Phylogenetic groups, the ST131 lineage, and virulence-associated genes were identified using PCR-based assays. Antimicrobial susceptibility testing (AST) was performed using automated methods and extended-spectrum beta-lactamase (ESBL) production was confirmed using the double-disk synergy test. Colistin (COL) resistance was evaluated by Colistin Drop Test and PCR screening for the *mcr-1* (mobile colistin resistance gene 1). Biofilm formation was detected by the Tissue Culture Plate (TCP) method, whereas phenotypic virulence factors (VF) were assessed with Congo Red agar, hemagglutination, and hemolysis assays. **Results:** Phylogenetic groups B2 (43.8%) and D (26.7%), typically associated with extraintestinal infections, were the most frequent. The high-risk clone B2-ST131 was detected in 6.7% of isolates. Biofilm production was observed in 92.4% of the isolates, with curli fimbriae (87.6%) being the most frequently expressed VF. The highest resistance rates were observed for ampicillin (62.1%), ampicillin-sulbactam (39.8%), and trimethoprim-sulfamethoxazole (25.2%). Interestingly, 3.8% of isolates exhibited colistin resistance, despite the absence of the *mcr-1* gene. **Conclusions:** This study highlights the detection of MDR-UPEC isolates that showed strong resistance to fluoroquinolones and were ESBL producers with high virulence in Argentina, justifying future research encompassing genomic and epidemiological monitoring of local UPEC, which is essential for managing infections and developing new therapeutic and preventive measures.

## 1. Introduction

Urinary tract infections (UTIs) are among the most prevalent bacterial infections in humans, affecting approximately 400 million individuals worldwide annually [1]. Extraintestinal pathogenic *Escherichia coli* (ExPEC) is the leading cause of UTIs [2,3]. These *E. coli* isolates, collectively known as Uropathogenic *E. coli* (UPEC), contain different virulence factors (VFs), such as adhesins, invasins, toxins, and iron-acquisition systems, which contribute to the pathogenicity of the strains [4]. Furthermore, the rise in multidrug-resistant (MDR)-UPEC strains is currently causing significant challenges in the treatment of infected patients [5]. *E. coli* strains exhibit a pronounced phylogenetic structure characterized by eight phylogenetic groups A, B1, B2, C, D, E, F, and an *Escherichia* cryptic clade I [6]. These phylogroups differ in both phenotypic and genotypic traits, including antibiotic resistance profiles and growth rates [7,8]. While intestinal *E. coli* isolates are found in groups A and B1, most UPEC strains that cause extraintestinal infections are found in groups B2 or D [8]. Strains belonging to the B2 group are resistant to most β-lactam antibiotics, mediated by the production of extended-spectrum β-lactamases (ESBLs). Within the B2 group, *E. coli* sequence type 131 (ST131) is recognized as a significant worldwide emerging clone, characterized by the presence of numerous resistance genes and virulence factors [9]. ST131 has expanded rapidly since the early 2000s, displacing other lineages and creating a significant public health challenge due to its transmission capacity, persistence, and resilience [10]. Furthermore, a mobile genetic element, such as pColV, which encodes several virulence genes, has been studied for its role in the pathogenesis of ExPEC strains of human and avian origin, including ST131 strains [11,12,13]. Recently, colicin V-like plasmids (ColVLPs) have been identified as elements that amplify the convergence of resistance and virulence in ExPEC, and their presence has been associated with increased virulence and antimicrobial resistance [13].

A major aspect of the pathogenic potential of UPEC is its ability to form biofilms. Biofilms are complex bacterial communities that are enveloped within a self-produced extracellular polymeric matrix. Biofilms can significantly affect human health. The sessile lifestyle of biofilms offers significant survival advantages, notably by enhancing antimicrobial resistance (AMR) and promoting the persistence of recurrent and chronic infections [14]. Bacteria residing within biofilms can demonstrate up to a 1000-fold increase in resistance to antimicrobial agents compared to their free-floating planktonic counterparts [15,16].

The global rise and dissemination of antibiotic-resistant strains have intensified the difficulty of treating biofilm-associated infections, rendering current therapeutic strategies ineffective and costly [17]. Understanding the virulence of *E. coli* is of utmost importance in infectious disease research. To deepen the knowledge of UPEC infections, this study aimed to characterize the phylogenetic groups, virulence factors, and antimicrobial susceptibility of UPEC isolates from a population with UTIs in the metropolitan area of Buenos Aires (AMBA), Argentina.

## 2. Results

### 2.1. Phylogenetic Groups

A total of 105 *E. coli* isolates were collected between 2019 and 2022. Phylogenetic analysis of *E. coli* isolates using the quadruplex PCR assay revealed that they mainly belonged to phylogroups B2 (43.80%, n = 46) and D (26.67%, n = 28), followed by A (10.48%, n = 11), B1 (7.62%, n = 8), F (7.62%, n = 8), and C (0.95%, n = 1). No isolates were assigned to phylogenetic group E or cryptic clade I, and three isolates (2.86%) were untypable.

### 2.2. Expression of Virulence Factors

**Figure 1:**
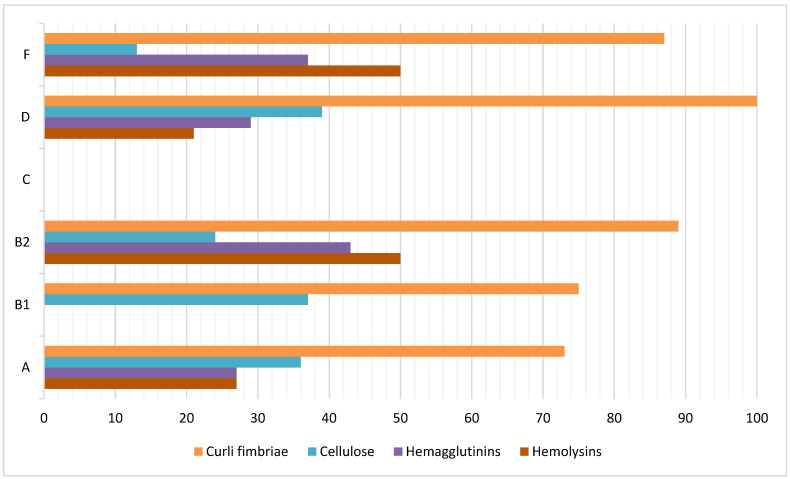
Distribution of virulence factor frequencies among the six phylogenetic groups found in the UPEC isolates.

The frequencies of virulence factor expression were as follows: fimbria curli (87.6%), hemolysins (38.1%), hemagglutinins (33.3%), and cellulose (30.5%). The isolate from phylogroup C did not express any of the virulence factors. Figure 1 shows the distribution of virulence factor frequencies among different phylogroups.

### 2.3. Biofilm Formation

**Figure 2:**
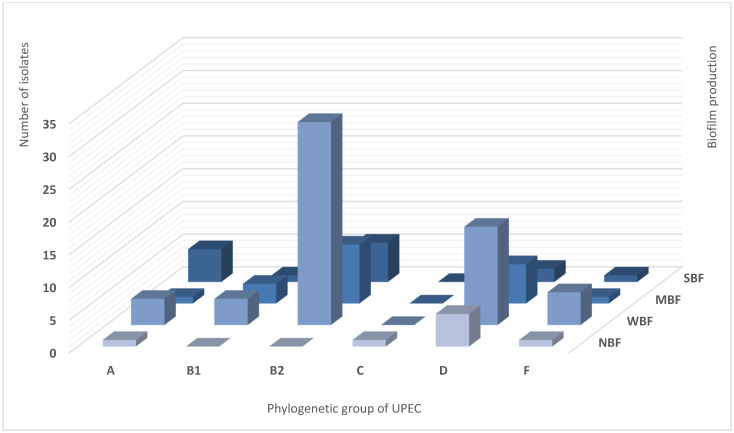
Number of UPEC isolates, phylogenetic groups and categories of biofilm production: Non-Biofilm Former (NBF), Weak Biofilm Former (WBF), Moderate Biofilm Former (MBF), Strong Biofilm Former (SBF).

Biofilm was detected in 92.4% (n = 97) of UPEC samples. The average biofilm amount (OD) was 0.63± 0.5. Bacterial isolates were categorized as Weak Biofilm Formers (WBF; 57.1%), Moderate Biofilm Formers (MBF; 21.0%), Strong Biofilm Formers (SBF; 14.3%), and Non-Biofilm Formers (NBF; 7.6%) (Table 1). The NBF isolates belonged to phylogenetic groups A, C, D, and F. Regarding biofilms, phylogroup B2 was prevalent in all categories: WBF (51%), MBF (38%), and SBF (39%). Of a total of 8 NBF isolates, 64% belonged to phylogroup D, and no isolates from group B2 were detected in this category. Figure 2 shows the number of UPEC isolates, phylogenetic groups, and biofilm production categories.

### 2.4. Virulence Genes

**Figure 3:**
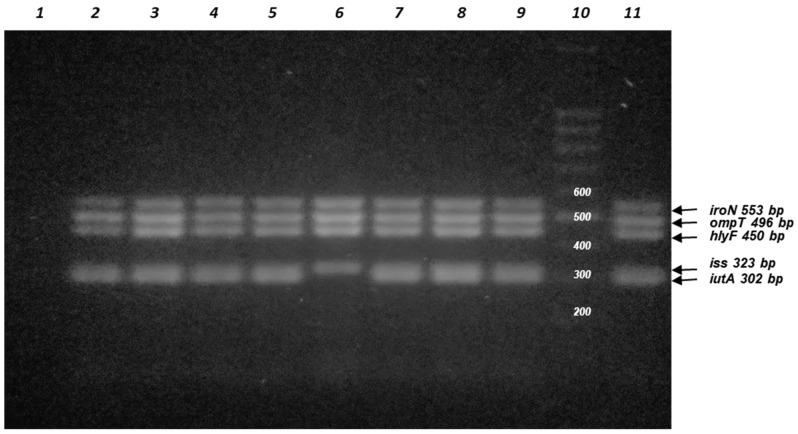
Agarose gel electrophoresis of PCR-amplified products for detection of five *E. coli* ColV plasmid virulence genes: *iutA* (aerobactin siderophore receptor), *hlyF* (outer membrane vesicle production), *iss* (increased serum survival), *iroN* (Salmochelin siderophore receptor), and *ompT* (Outer membrane protease) in UPEC isolates. Line 1: negative control (no DNA template was added); Line 2: U007; line 3: U015; Line 4: U025; Line 5: U028; Line 6: U062; Line 7: U070; Line 8: U071; line 9: U096; Line 10: DNA ladder (100 bp); Line11: isolate APEC A14 positive control (sequenced isolate that contains the 5 VG (unpublished results).

In addition to the expression of the identified virulence factors, five virulence genes- *iutA* [aerobactin siderophore receptor], *iss* [increased serum survival], *hlyF* [upregulates outer membrane vesicle production], *ompT* [outer membrane protease], and *iroN* [salmochelin siderophore receptor]- were examined. These genes are associated with pColV-like plasmids and are important markers for analyzing the virulence profile, because they are linked to increased virulence of ExPEC isolates [12,13,18,19]. In particular, the *hlyF* gene is associated with increased production of outer membrane vesicles, possibly contributing to the release of cytolethal distending toxin and other chemicals involved in ExPEC pathogenesis [20,21]. Eight UPEC isolates contained at least four of the five genes tested. These isolates were assigned to phylogroups B2, F, A, and B1 at 50%, 25%, 12.5%, and 12.5%, respectively. These genes were absent in the other groups (C and D). Figure 3 shows PCR-amplified products of five *E. coli* ColV-like plasmid virulence genes.

### 2.5. Antimicrobial Susceptibility

The antimicrobial susceptibility of 105 UPEC isolates was tested against 17 antimicrobial compounds (six classes). The antimicrobials with the highest frequency of resistance (R) were AMN (62.1%), followed by TMS (25.2%) and CIP (23.5%). The R frequencies for beta-lactams with beta-lactamase inhibitors were 39.8% (AMS) and 11.7% (TAZ). The R frequencies for third-generation cephalosporins (CAZ/CTX) were 12.6%. The remaining ATM (IMP, MER, LEV, GEN, NIT, and COL) had R values of <5% of the isolates. All isolates were sensitive to ERT and AKN. In particular, four isolates showed resistance to COL by phenotypic methods (3.8%), but none harbored the *mcr-1* gene.

**Figure 4:**
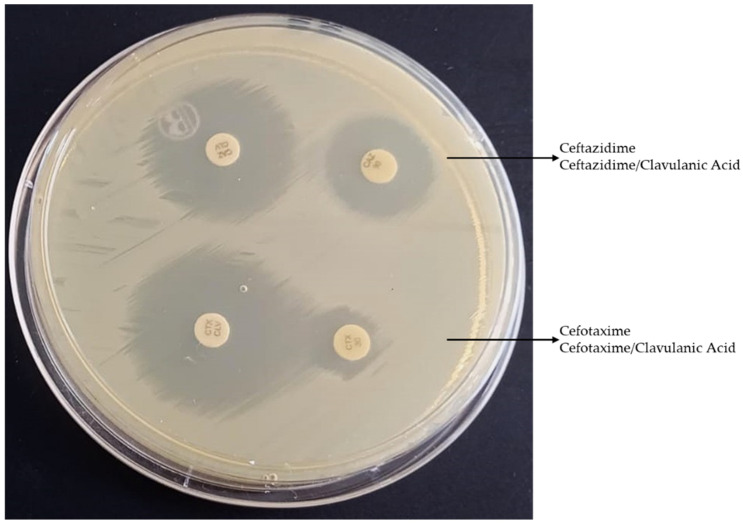
Phenotypic detection of *E. coli* strains that produce extended-spectrum beta-lactamase (ESBL) using the double-disc synergy test (DDST).

**Figure 5:**
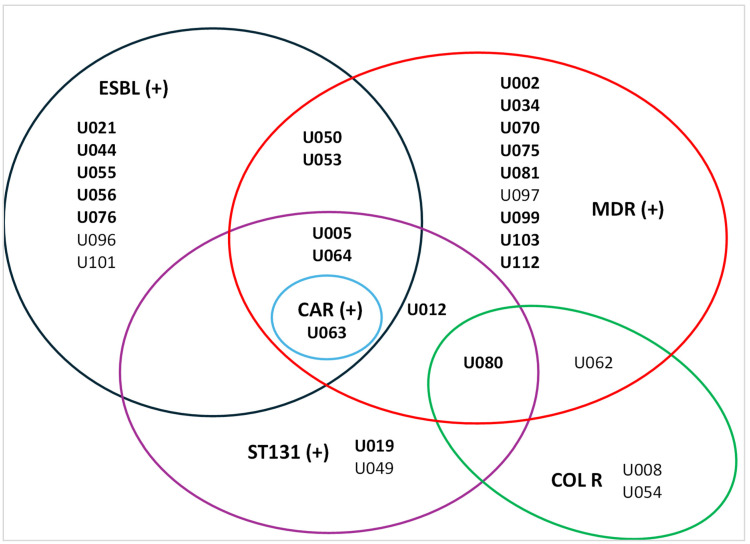
Antimicrobial resistance profiles of UPEC isolates. Extended spectrum beta-lactamase (ESBL), multidrug resistance (MDR), carbapenemase (CAR), and colistin resistance (COL R). Number of isolates in bold type indicate ciprofloxacin resistance (CIP R).

The global ESBL-UPEC rate was 11.5%; 95% CI [0.068, 0.193] (Figure 4). The most frequent ESBL producers were observed in phylogenetic group B2, followed by A, B1, D, and F. The global rate of FQ-R was 23.5%, whereas ST131 isolates showed higher resistance (85.7%). FQ-R was observed in phylogenetic groups B2, A, and D, whereas B1, C, and F showed no FQ-R. The global rate of MDR-UPEC was 16.5%; 95% CI [0.106, 0.249], with phylogenetic group B2 being the most common, followed by D, A, and B1. Figure 5 illustrates the resistance profiles of UPEC isolates.

### 2.6. Detection and Characteristics of ST131

**Figure 6:**
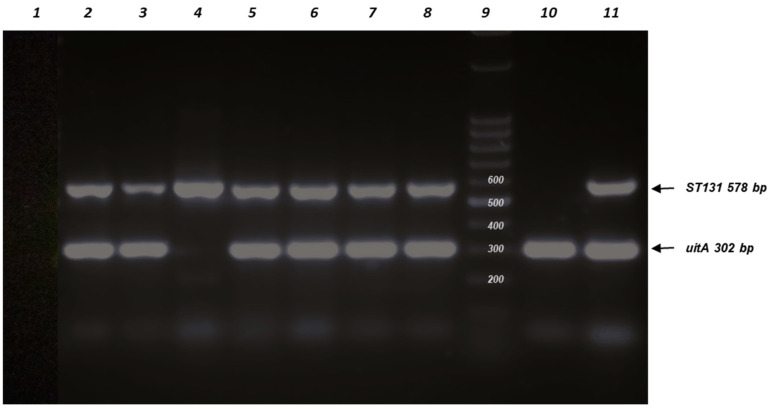
Agarose gel electrophoresis of PCR-amplified products for detection of *E. coli* ST131 isolates. Line 1: negative control (no DNA template was added); Line 2, isolate U005 (ST131+, *iutA*+); Line 3, isolate U012 (ST131+, *iutA*+); Line 4, isolate U019 (ST131+, *iutA*−); Line 5, isolate U049 (ST131+, *iutA*+); Line 6, isolate U063 (ST131+, *iutA*+); Line 7, isolate U064 (ST131+, *iutA*+); Line 8, isolate U080 (ST131+, *iutA*+); Line 9, DNA ladder (100 bp); Line 10, isolate APEC A14 ST117 (ST131−, *uitA*+) control; Line 11, (ST131+, *iutA*+) control.

Among the UPEC isolates, ST131 isolates were identified because they represent a high-risk pandemic lineage, frequently multidrug-resistant (MDR), and cause serious infections, such as UTIs and bacteremia. Their detection allows for the prediction of antimicrobial resistance (AMR), such as FQs and ESBL enzymes [22,23,24]. The gene *iutA* (aerobactin receptor) is considered a virulence factor used to define ExPEC status and has been reported to be frequently present in *E. coli* ST131 isolates [22]. Seven ST131 isolates (6.7%; 95% CI [0.033, 0.131]) were detected by PCR among the UPEC isolates, and all but one of the ST131 isolates were *iutA* positive (85.7%). Figure 6 shows PCR-amplified products for the detection of *E. coli* ST131 isolates and *iutA* genes.

**Figure 7:**
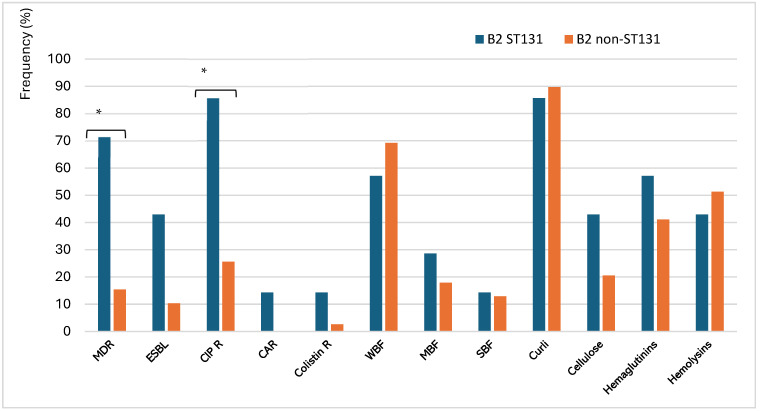
Frequency of virulence factors and antimicrobial resistance of ST131 and non-ST131 UPEC isolates (* *p* < 0.05). Multi-drug resistance (MDR), Extended-Spectrum Beta-Lactamase (ESBL), Ciprofloxacin resistance (CIP R), Carbapenemase (CAR), Colistin resistance (COL R); Weak Biofilm Former (WBF), Moderate Biofilm Former (MBF), Strong Biofilm Former (SBF).

In terms of AMR, ST131 accounted for 29.4% of MDR-UPEC and 25% of FQ-R isolates. Significant differences were noted between B2 ST131 and B2 non-ST131 isolates concerning MDR (*p* = 0.00544) and CIP R (*p* = 0.00865). Conversely, no significant associations were found between these groups for ESBL, carbapenemase, and COL R (*p* > 0.05). Regarding virulence factors, both groups showed weak biofilm formation (WBF), and no significant differences were observed in the biofilm-forming ability for all categories (*p* > 0.05) (Table 2). Likewise, all virulence factors, including curli fimbriae, cellulose, hemagglutinins, and hemolysins, were uniformly distributed, indicating that fundamental pathogenicity was preserved in this lineage. Figure 7 illustrates the virulence profiles of ST131 and non-ST131 isolates.

## 3. Discussion

The phylogenetic classification of UPEC isolates is relevant for clarifying the relationship between strains and diseases. Global epidemiological data indicate that phylogenetic group B2 is the most prevalent lineage, with clinical isolation rates typically ranging between 40% and 65%, a phenomenon attributed to its extensive repertoire of virulence factors [25]. In this study, the prevalent phylogroup was B2 (43.8%), whereas the other groups (D, A, B1, F, and C) were detected at lower frequencies. These results are consistent with those of several previous studies. For example, a systematic review published in 2022 noted that most UPEC isolates were classified into phylogenetic group B2 in the populations of Brazil, China, Denmark, Ethiopia, France, Iran, Pakistan, Romania, and South Korea [8,26,27]. The predominance of phylogroup B2 in UPEC has also been reported in Iraqi outpatients (34%) and Chinese inpatients (52%) with urinary infections [28,29]. In Latin America, recent genomic studies have revealed a high prevalence of phylogroup B2 in clinical isolates from Argentina, Brazil, Colombia, Mexico, Paraguay, and Peru [30]. Phylogenetic group D is recognized as the second most significant group, accounting for approximately 15–25% of cases, whereas groups A and B1 are primarily associated with commensal strains and exhibit significantly lower frequencies of extraintestinal infectious processes [31]. In our UPEC isolates, phylogenetic group D accounted for 26.67%, making it the second most prevalent group. Our results are in accordance with the values of this phylogroup reported in Honduras (21%), China (28%), Iran (20–26%), and Colombia (25.3%) [8,27,29,32,33]. Variations in the occurrence of phylogenetic groups may be attributed to host genetic factors, site of infection, geographical distribution, methodological differences, origin of isolates, or variations in sample size [8,22].

UPEC isolates were phenotyped for several virulence factors. The most highly expressed virulence factor was curli fimbriae, whereas hemolysins, hemagglutinins, and cellulose were detected at lower frequencies. These results are consistent with those of our previous study. The high prevalence of curli fimbriae in *E. coli* isolates is consistent with values observed in other studies. For example, our previous study found a frequency of expression of this adherence factor of 92.3% in UPEC isolates [34]. Similarly, these results are in line with Milanov et al. [35], who reported the presence of curli fimbriae in all *E. coli* isolates. In contrast, Schiebel et al. [36] reported curli expression lower than 60%. Curli fimbriae, surface proteins crucial for *E. coli* biofilm development, specifically interact with host matrix proteins, such as fibronectin, laminin, and plasminogen. This interaction initiates the adherence and invasion of host cells, activates the immune system, and promotes bacterial aggregation and biofilm formation [37]. In our study, approximately one-third of the isolates produced hemagglutinins, hemolysins, or cellulose, which is consistent with findings from several studies [38,39]. However, other researchers have reported varying values [40,41,42,43]. These observed variations in the expression of virulence factors may be attributed to several factors, including the virulence of the isolate and the clinical characteristics of the patient, such as age, site of infection, comorbidities, antimicrobial consumption, and recurrence of infectious disease.

Biofilm-related diseases significantly contribute to increased morbidity and mortality rates among patients and impose a considerable economic burden, including substantial healthcare costs and prolonged hospital stays. The development of organized bacterial communities represents a paradigm shift in the pathogenesis of infectious diseases, underscoring the need for novel strategies to detect and combat biofilm-associated infections [34]. Worldwide, biofilm formation in *E. coli* varies between 13.3% and 99% [44]. In our study, biofilms were identified in 92.4% of the UPEC samples, and phylogroup B2 was prevalent in all categories of biofilm formation. A recent study by our group showed similar findings, with biofilm production observed in 100% of all UPEC isolates [34]. Consistent with our results, several studies have reported biofilm production in over 90% of UPEC isolates [36,45,46,47,48]. In contrast, several reports have indicated a lower frequency of biofilm production by UPEC [39,41]. The results of our study indicated that among the biofilm-producing isolates, 57.1% were weak biofilm producers, 21% were moderate, 14.3% were strong, and 7.6% were non-biofilm formers. The WBF category was also predominant for UPEC isolates from India (36%), Thailand (56%), and Iran (72%) [49,50,51]. Similarly, Naziri et al. [47]. studied UPEC isolates from outpatients and inpatients and found that weak producers were the most prevalent. However, several studies have reported differences. Two recent studies on UPEC reported that the main biofilm category was strong [40,48]. Other authors have indicated that the moderate category is prevalent in UPEC isolates [52,53]. Finally, the absence of biofilms (NBF) in UPEC isolates has been reported in several studies. For example, this category has been found in Iran (9%), India (28%), and Slovenia (44%) [49,54,55]. Although most *E. coli* isolates formed biofilms, there was significant variability among them. This variation could be attributed to factors such as the source of isolation (urine, feces, or environment), virulence of isolate (commensal or pathogenic), underlying pathology, previous treatment, clinical evolution of infection, and patient status (ambulatory or hospitalized).

The antimicrobials with the highest frequency of resistance were AMN (62.1%), followed by AMS (39.8%), TMS (25.2%), and CIP (23.5%). These rates are consistent with those of other studies. A global study on UPEC isolates showed that the resistance to AMS, CIP, and TMS always remained below 40% in developed countries, while in developing countries, it increased to 50% [52]. Moreover, our findings align with those of previous studies conducted in Argentina. For instance, AMR values for UPEC were reported by the Argentine Consensus on Urinary Infection in 2020. This report revealed average resistance rates of 58% for AMN, 30% for TMS, and 16% for CIP [56]. Similarly, research conducted in the Mendoza province indicated UPEC resistance to AMS and CIP at rates of 38.1% and 22.2%, respectively [57]. Finally, WHONET revealed that 12.7% of UPEC isolates in Argentina were resistant to third-generation cephalosporins, which is in close agreement with the results of our study.

The emergence of colistin resistance, mediated by chromosomal mutations and the dissemination of plasmid-borne *mcr* genes, constitutes a significant challenge to the effective treatment of infections caused by multidrug-resistant Gram-negative bacteria. The identification of four isolates (3.81%) showing phenotypic resistance to colistin using the Colistin Drop Test (CDT) led to the search for the *mcr-1* gene, as it is the most common plasmid-mediated colistin resistance gene in clinical infections [58,59,60]. Since its identification in 2015 in *E. coli* from pigs in China, *mcr-1* has disseminated globally, with reports across Europe, Asia, Africa, and the Americas [58,59,60]. However, all isolates were PCR-negative for the *mcr-1* gene. This finding requires further investigation to elucidate the mechanisms underlying colistin resistance. In addition to *mcr-1*, other plasmid-mediated *mcr* variants (*mcr-2* to *mcr-10*), as well as chromosomal mutations that alter the lipid A component of lipopolysaccharides (particularly those involving the PmrA/PmrB and PhoP/PhoQ two-component regulatory systems) may contribute to COL R [59,60,61]. Other less frequent mechanisms include overexpression of efflux pumps or capsular polysaccharides.

In this research, the ESBL-UPEC and MDR-UPEC rates were 11.5% and 16.5%, respectively. Phylogenetic group B2 was prevalent in both categories. These findings are consistent with those of previous studies. A global surveillance study conducted between 2004 and 2022, which examined AMR values from 64 countries and over 113,000 patients, reported that global ESBL-UPEC and MDR-UPEC were detected in 18.79% and 26.6% of cases, respectively. In particular, this study showed a prevalence of 11.78% for ESBL-UPEC and 15.48% for MDR-UPEC isolates in Latin America [62]. In Asia, the proportion of ESBL-UPEC isolates among patients with acute cystitis in Japan was 4.1%, whereas in Korea and China, it was 10.8% and 38.07%, respectively [63,64,65]. In Europe, the frequency of ESBL-UPEC varies from 2% to 24% [66,67,68]. In the Americas, the frequency was 12% in Argentina, 14.1% in Canada, 16.0% in the United States, and 31.3% in Mexico [69,70,71].

The predominance of the B2 phylogenetic group among ESBL and MDR UPEC isolates is in accordance with reports from several countries, including Brazil, Iran, Romania, and Thailand [26,72,73,74]. B2 phylogenetic group predominance between ESBL and MDR UPEC isolates was in line with reports from several countries like Brazil, Iran, Romania, and Thailand [26,72,73,74]. Geographical differences likely explain most of the variations in these categories between studies. However, the criteria for including or excluding isolates also play a crucial role, potentially leading to over- or underestimation of antimicrobial resistance in community-acquired infections [22,62,75].

In addition to the expression of the identified virulence factors, we examined five virulence genes: *iutA* (aerobactin siderophore receptor), *iss* (increased serum survival), *hlyF* (upregulates outer membrane vesicle production), *ompT* (outer membrane protease), and *iroN* (salmochelin siderophore receptor) in our UPEC samples. In particular, the *hlyF* gene is associated with increased production of outer membrane vesicles, possibly contributing to the release of cytolethal distending toxin and other chemicals. It has recently been reevaluated for its role in uropathogenesis, as it has been associated with more severe cases of UTI in humans [18,20,21]. These genes are associated with pColV-like plasmids (ColVLP), which are important markers assessing the virulence profile because they are linked to increased virulence in ExPEC isolates [12,13,18,19]. Therefore, we searched for the presence of ColVLP in our isolates using the five genes proposed by Johnson et al. [12]. Eight UPEC isolates carried all five genes tested, except for one, which contained only four, and were therefore categorized as ColVLP(+). None of these isolates was ST131. This result is consistent with the observation of high rates of ColVLP carriage in Clade B ST131 strains, which are strongly associated with foodborne infection compared with Clade A and Clade C ST131 strains, which are primarily derived from human infection [58].

The ST131 clone is a major cause of urinary tract infections, bacteremia, and other severe infections in both community and hospital settings. Its widespread success is due to a combination of high AMR, particularly against FQ and beta-lactams, and the acquisition of VFs that enable it to colonize and persist in the human gastrointestinal tract. In 2014, Nicolas-Chanoine et al. [22] published a comprehensive review of *E. coli* ST131. The authors highlighted the varying prevalence across different geographical regions and for different infection types, reporting a wide range of infection frequencies (3–98%) with this pandemic clone. In this study, ST131 was found in approximately 7% of UPEC isolates. Our results are consistent with those of other studies. For example, a recent work in Iraq reported a prevalence of ST131 of UPEC isolates of 7% [76]. Lower values of ST131 were observed in cystitis (6%) and pyelonephritis cases (11%) in the Australian population, in a pediatric population in the US (10%), and in health centers in Spain (12%) [22]. Several studies conducted in Asia have estimated the frequency of ST131. One of these studies was conducted in an outpatient population with UTIs in Taiwan and showed a prevalence of 11–17% [77]. Other studies conducted in Thailand, Singapore, Malaysia, and Western Asia showed a prevalence of UPEC of approximately 20–24% [78]. In contrast, frequencies exceeding 30% have been reported. For example, a recent study published in 2025 reported that the most prevalent clone in Latin America was ST131 (42.7%) [30]. Similarly, ST131 was detected in 39.1% and 47.9% of the isolates in Spain and France, respectively [79]. The variable frequency of ST131 may be attributed to several factors, including the source of infection, whether community-based or healthcare-associated, and the clinical progression of the infection [80].

ST131 is a widespread extraintestinal pathogenic *E. coli* that has attracted significant public attention because of its increasing AMR and high pathogenicity. ST131 isolates are particularly concerning because of their frequent resistance to multiple commonly prescribed antibiotics. Our study revealed that ST131 was identified in 25% of ESBL-UPEC isolates. These findings align with previous research reporting ST131 frequencies of <30% in ESBL-UPEC isolates in countries such as France (21%), Spain (22%), Canada (23%), and Mexico (25%). Conversely, several studies focusing on ESBL-UPEC have shown ST131 frequencies exceeding 30%, with reports from the Czech Republic (44%), Japan (52%), Canada (53%), the United States (64%), Israel (85%), and Iran (95.2%) [22]. In terms of multi-resistance, 71.4% of our ST131 isolates were MDR. Similar results have been reported in recent years. For example, two recent studies conducted in patients with UTI in Iran and Croatia reported a frequency of MDR-ST131 of 72% and 80%, respectively [22,26,27,81,82].

In recent decades, FQ resistance has increased significantly in several European countries, as well as in Asia and South America. However, a global review of UPEC reported that resistance was markedly higher (50–85%) in developing countries than in developed countries (5–30%) [52]. Consistent with these findings, our UPEC isolates exhibited a CIP resistance rate of 23.5%, although ST131 showed an even higher value of 85.7%. This result matches several reports. For example, the prevalence of ST131 among FQ-UPEC isolates was particularly high in Iran (70%), Spain (70%), Taiwan (72.1%), and the US (78%) [22,27,78].

The misuse and inadequate regulation of broad-spectrum antibiotics have significantly contributed to the rise in AMR, particularly in low- and middle-income countries [52]. In this study, significant differences were noted between ST131 and non-ST131 isolates concerning MDR and FQ R. In contrast, the analysis did not reveal any significant correlations between the groups regarding ESBL and COL resistance rates. Similarly, biofilm formation and virulence factors were evenly distributed, suggesting that basal pathogenicity is conserved within this lineage. A comparative analysis of ST131 and B2 non-ST131 isolates revealed distinct specializations in their antimicrobial susceptibility profiles. The most significant finding was the association of ST131 with MDR and FQ R. In contrast, no statistical differences were found in virulence factors, suggesting that both groups shared a similar set of virulence traits characteristic of the B2 group, which is inherently pathogenic.

The unique distribution of virulence and resistance characteristics in ST131, as compared to non-ST131 B2 isolates, is likely the result of complex evolutionary compensation. Genetically, ST131, especially the H30-Rx sublineage, has acquired mutations that mitigate the fitness costs usually associated with MDR. This adaptation allows for the stable maintenance of large IncF plasmids that contain both ESBL genes and specific virulence factors. Metabolically, ST131 shows an improved capacity to obtain nutrients in the nutrient-poor environment of the urinary tract, using specialized iron-uptake systems (e.g., iutA) and efficient D-serine metabolism, which gives it a competitive advantage over other phylogroups. Ecologically, these traits result in a superior ability for clonal expansion and long-term colonization of the intestinal reservoir, aiding its rapid spread in both hospital and community settings. As a result, while non-ST131 isolates may concentrate on either high virulence or high resistance, ST131 combines both, reinforcing its position as a leading uropathogen.

The findings of this study make a significant contribution to the field of medicine by shedding light on the virulence profile of uropathogenic *E. coli*. However, this study has certain limitations. One of these was the small number of isolates and the absence of medical data regarding the types of urinary infections (whether uncomplicated or complicated, community-acquired, or nosocomial), the antimicrobial therapies prescribed, or any prior hospitalizations of the patients. In addition, the number of virulence genes studied was small. Moreover, the study was conducted in vitro, focusing solely on biofilms formed on polystyrene surfaces and static cultures, which may not accurately reflect the characteristics of UPEC biofilms in the human body. Finally, the relatively small sample size and restricted temporal and geographical scope (AMBA) of the isolate collection may limit the extrapolation of these findings to other regions of the country.

The results of this research constitute a relevant contribution to microbiology and provide new information on the virulence profile of local uropathogenic *E. coli*. The prevalent phylogenetic group was B2, whereas the other groups (D, A, B1, F, and C) were detected at lower frequencies. Regarding AMR, the rates of ESBL-UPEC and MDR-UPEC were 11.5% and 16.5%, respectively. Mobile colistin resistance is the primary mechanism for COL R worldwide. The global spread of *mcr* genes was attributed to the high selective pressure of COL used in livestock and the acquisition of mcr by low-copy-number and highly conjugative plasmids. The finding of resistance to COL in the absence of *mcr-1* in four isolates is relevant and could suggest chromosomal resistance mechanisms or new mobile genes. Biofilm production was detected in 92.4% of the isolates, and weak biofilms (WBF) were prevalent. The most highly expressed virulence factor was curli fimbriae, whereas hemolysins, hemagglutinins, and cellulose were detected at lower frequencies. ST131 isolates were detected in our samples that showed 71.4% MDR, 25% ESBL, and 85.7% CIP R. Significant differences were noted between ST131 and B2 non-ST131 isolates concerning MDR and CIP R.

The absence of this resistance mechanism in other UPEC isolates implies that this clone may function as a crucial reservoir for last-line resistance. Conversely, no significant associations were found between the groups for ESBL and COL R rates. Similarly, biofilm formation and virulence factors were evenly distributed, suggesting that the basal pathogenicity is conserved within this lineage. The results presented here underscore the need to implement continuous antimicrobial surveillance, given that the finding of Col R and ST131 MDR clones poses a significant public health risk in Argentina. Further studies on UPEC, including genomic and epidemiological surveillance, will be crucial for improving infection management and the development of preventative strategies.

## 4. Materials and Methods

### 4.1. Collection of Bacterial Isolates

This was an observational, descriptive, retrospective, cross-sectional study. A collection of 105 non-duplicate UPEC isolates was investigated. The isolates were collected between 2019 and 2022 as part of routine microbiology services in the metropolitan region of Buenos Aires (AMBA). The isolates were identified as *E. coli* using manual and automated methods (Phoenix, Becton Dickinson, Franklin Lakes, NJ, USA) and preserved in brain heart infusion broth (Britania Lab, Buenos Aires, Argentina) containing 20% glycerol. This study exclusively employed bacterial isolates that were independent of any information and did not involve human-derived material, tissue, or data. The researchers conducted the study in accordance with the standards of good clinical practice, the Declaration of Helsinki with all its amendments, and applicable regulations. The standards of confidentiality were upheld in accordance with Law No. 25,326 (Personal Data Protection Act) and Articles 51 and 52 of the Argentine Civil and Commercial Code. The information collected is confidential, and the names of the participants in this study do not appear in any records or are disclosed. The confidentiality of the research was observed by assigning an internal alphanumeric code that allowed the permanent dissociation of the patient’s information.

### 4.2. Phylogenetic Group Affiliation and ST131

DNA was extracted by boiling (100 °C for 5 min), and bacterial lysates were centrifuged at 11,000× *g* for 3 min. The supernatant was used as a template for PCR reactions. The new Clermont *E. coli* phylogenetic groups (*E. coli* sensu stricto A, B1, B2, C, D, E, F, and *E. coli* clade I) were determined using the quadruplex PCR by amplifying four DNA markers (*arpA*, *chuA*, *yjaA*, and TSPE4.C2), duplex A/C PCR, and duplex D/E phylogenetic group-specific PCRs, as described by Clermont et al. [6]. *E. coli* ATCC 25922 [*arpA* (−), *chuA* (+), *yjaA* (+), TspE4.C2 (+)] was used as a positive control. The amplified products were analyzed by standard gel electrophoresis on 1.8% agarose gels (Biodynamics, Argentina) with SYBR^®^ Safe DNA Gel Stain (Life Technologies, Carlsbad, CA, USA) and interpreted according to the guidelines for phylogenetic group assignment [6]. The ST131 marker gene was detected as described by Johnson et al. [83]. The primer sequences and sizes of the PCR products are detailed in Supplementary Table S1.

### 4.3. Expression of Virulence Factors

The expression of curli fimbriae and cellulose production was evidenced by culture on Congo Red Agar [84]. Macroscopic appearance and pigmentation of the colonies were used to classify the isolates into morphotypes [85]. The expression of hemagglutinins was revealed by the agglutination, as described by Molina et al. [34]. Hemolysin expression was detected on Blood Agar. For each isolate, the bacterial suspension was inoculated on agar supplemented with sheep blood (5–10%) (Britania, Argentina) and incubated for 24 h. Hemolysis was identified by the presence of a clear zone around the colony [34].

### 4.4. Biofilm-Forming Ability

To estimate biofilm production, each isolate was incubated in sterile 96-well polystyrene microtiter plates with Luria–Bertani (LB) broth. Briefly, each well was inoculated with a 12-h LB liquid culture previously adjusted to an OD600 of 0.2 (Beckman Spectrophotome, Brea, CA, USA) and incubated for 24 h. After washing the plates with sterile phosphate-buffered saline, sessile cells were dried at 60 °C and stained with crystal violet solution (Merck, Buenos Aires, Argentina). The stain absorbed by the biofilms was dissolved in ethanol (96°), and the optical density was measured at OD590 nm using a microtiter plate reader (Bio-Rad Laboratories, Hercules, CA, USA) according to Molina et al. [34]. *E. coli* ATCC 25922 was used as a positive control for the assay, and LB broth without bacteria was the negative control. Each assay was performed in triplicate in two independent replicates. For the negative control, 12 wells per plate were inoculated with 200 μL of LB broth. The biofilm formation capacity for each isolate was estimated based on Stepanović et al.’s [12]. For this purpose, we determined the cut-off optical density (ODc) to be three SDs above the mean OD of the negative control. The biofilm-forming bacteria were categorized based on OD values: Non-Biofilm Former, NBF (OD ≤ ODc); Weak Biofilm Former, WBF (ODc < OD ≤ 2 ODc); Moderate Biofilm Former, MBF (2 ODc < OD ≤ 4 ODc); and Strong Biofilm Former, SBF (OD > 4 ODc) [36].

### 4.5. Antimicrobial Susceptibility Profile

In accordance with the Clinical and Laboratory Standards Institute guidelines (CLSI), the antimicrobial susceptibility of the isolates was assessed using manual and automated methods (Phoenix, Becton Dickinson). The results were categorized as sensitive (S) or resistant (R) [86]. The susceptibility of the *E. coli* isolates to 17 antimicrobials (ATM) belonging to six classes was tested: Amoxicillin (AMN), Amoxicillin/Sulbactam (AMS), Piperacillin/Tazobactam (TAZ), Cephalexin (CEF), Cefotaxime (CTX), Ceftriaxone (CAZ), Cefepime (FEP), Imipenem (IMP), Meropenem (MER), Ertapenem (ERT), Ciprofloxacin (CIP), Levofloxacin (LEV), Gentamicin (GEN), Amikacin (AKN), Trimethoprim/Sulfamethoxazole (TMS), Nitrofurantoin (NIT) and Colistin (COL). Resistance to NIT was evaluated using the Kirby-Bauer disc diffusion method, and resistance to COL was detected using the Colistin Drop Test (CDT). Phenotypic detection of *E. coli* strains that produce extended-spectrum beta-lactamase (ESBL) was conducted using the double-disc synergy test (DDST) in accordance with the CLSI guidelines [86]. Multidrug resistance (MDR) was defined according to Magiorakos et al. [87].

### 4.6. Detection of Virulence Genes and mcr-1 Gene

The virulence genes examined included siderophores (*iutA*, *iroN*), outer membrane vesicle production (*hlyF*), increased serum survival (*iss*), and outer membrane proteins (*ompT*). These genes are linked to Colicin V plasmids (pColV) and were amplified using pentaplex PCR, as described by Johnson et al. [83]. 

The plasmid-mediated colistin resistance (*mcr-1*) gene was detected using a method described by Liu et al. [88]. Briefly, amplification of bacterial DNA was performed on a total volume of 25 μL containing 5 μL of lysate, 150 ng of each primer, 200 μM of each dNTP (Promega, Fitchburg, WI, USA), GoTaq 1X reaction buffer, 1.5 μM MgCl_2_, and 1 U of GoTaq DNA polymerase (Promega, WI). The PCR conditions were as follows: initial denaturation (94 °C for 5 min), 35 cycles (94 °C for 30 s, 52 °C for 30 s, and 72 °C for 30 s), followed by a final extension (72 °C for 5 min). PCR analyses were performed on the samples using a Px2 Thermal Cycler (Thermo Fisher Scientific, Waltham, MA, USA). (Supplementary Table S1). PCR products were resolved on 1.8% agarose gels (Biodynamics, Argentina) and stained with SYBR^®^ Safe (Life Technologies, USA). The sizes of the amplified products were compared with those of positive controls and a DNA molecular marker (100 bp DNA ladder).

### 4.7. Statistical Analysis of Data

Descriptive statistical analyses were conducted using the Statistical Package for the Social Sciences (SPSS v.20.0). Qualitative variables were presented as frequencies, while quantitative variables were shown as means and standard deviations. The 95% CI for key prevalence estimates was calculated using the Wilson Score Interval. Univariate analyses were performed with the Chi-Square or Fisher’s exact tests. *p*-values below 0.05 were considered significant.

## Figures and Tables

**Table 1:** Average OD of biofilm production in UPEC isolates.

**BF Category**	$\bar{X}\pm S$ *	**95% CI**
Non-Biofilm Former (NBF)	0.15 ± 0.03	[0.13, 0.18]
Weak Biofilm Former (WBF)	0.38 ± 0.15	[0.34, 0.42]
Moderate Biofilm Former (MBF)	0.83 ± 0.25	[0.72, 0.94]
Strong Biofilm Former (SBF)	1.60 ± 0.5	[1.32, 1.87]

* Average and Standard Deviation, OD: Optical Density. CI: confidence interval.

**Table 2:** Comparation between B2 ST131 versus B2 non-ST131.

Variable	*p*	OR	95% IC
MDR	0.00544 *	11.3385	[2.041, 62.996]
CIP R	0.00865 *	10.3623	[1.543, 69.570]
ESBL	0.06005	6.1358	[1.113, 33.816]
COL R	0.28406	5.9231	[0.531, 66.013]
CARBA	0.15217	18.2308	[0.668, 497.477]
WBF	0.66667	0.5844	[0.124, 2.749]
MBF	0.60909	1.9697	[0.362, 10.704]
SBF	1.00000	1.4476	[0.197, 10.594]
Curli fimbria	1.00000	0.5493	[0.072, 4.175]
Cellulose	0.33284	2.8824	[0.588, 14.121]
Hemagglutinins	0.68159	1.8312	[0.395, 8.475]
Hemolysins	0.70701	0.7398	[0.160, 3.410]

MDR: Multidrug resistance, CIP R: Ciprofloxacin resistance, ESBL: Extended-spectrum beta-lactamase, COL R: Colistin resistance, CARBA: Carbapenemase, WBF: Weak Biofilm Former, MBF: Moderate Biofilm Former, SBF: Strong Biofilm Former. * (*p* < 0.05).

## Data Availability

The original contributions presented in this study are included in the article/Supplementary Material. Further inquiries can be directed to the corresponding author.

## Supplementary Materials

The following supporting information can be downloaded at: https://www.mdpi.com/article/10.3390/antibiotics15040350/s1, Table S1: Primer sequences used in the PCR assay for the detection and identification of virulence genes. Primers used for the detection of *E. coli*. PCR primers sequence, target, and amplicon size used in this study.
