# Supplementary material for: Phylogenetic Groups, Virulence Factors, and Antimicrobial Susceptibility of Escherichia coli Associated with Urinary Tract Infections from a Metropolitan Area of Buenos Aires, Argentina
Source: Antibiotics (Basel). 2026 Mar 29;15(4):350. doi: 10.3390/antibiotics15040350 (PMC13113254; doi:10.3390/antibiotics15040350)
Supplement: Supplementary file 1 [file antibiotics-15-00350-s001.zip › antibiotics-4204079-supplementary.pdf]

Supplementary Material

Manuscript: Phylogenetic groups, virulence factors, and antimicrobial susceptibility of Escherichia coli associated with urinary tract infections from a metropolitan area of Buenos Aires, Argentina

Table S1. Primer sequences used in the PCR assay for the detection identification of virulence genes. Primers used for the detection of *E. coli*. PCR primers sequence, target, and amplicon size used in this study.

Name	Sequence (5′– 3′)	Target (reference)	Amplicon size (bp)
ST131_F<br>ST131_R	GTTCGACAAAATCCTCTCCG<br>GCACAACCAGACAAAGCAG	Divalent metal cation transporter	578
*CLR5*_F<br>*CLR5*_R	CGGTCAGTCCGTTTGTTC<br>CTTGGTCGGTCTGTAGGG	Plasmid-mediated colistin resistance	309
*hlyF*_F<br>*hlyF*_R	GGCCACAGTCGTTTAGGGTGCTTACC<br>GGCGGTTTAGGCATTCCGATACTCAG	outer membrane vesicle production	450
*iss*_F<br>*iss*_R	CAGCAACCCGAACCACTTGATG<br>AGCATTGCCAGAGCGGCAGAA	Episomal increased serum survival gene	323
*iroN*_F<br>*iroN*_R	AATCCGGCAAAGAGACGAACCGCCT<br>GTTCGGGCAACCCCTGCTTTGACTTT	Salmochelin siderophore receptor gene	553
*ompT*_F<br>*ompT*_R	TCATCCCGGAAGCCTCCCTCACTACTAT<br>TAGCGTTTGCTGCACTGGCTTCTGATAC	Outer membrane protease	496
*iutA*_F<br>*iutA*_R	GGCTGGACATCATGGGAACTGG<br>CGTCGGGAACGGGTAGAATCG	Aerobactin siderophore receptor gene	302
